# A Randomized, Double‐Blind, Controlled Phase II Study of Foliglurax in Parkinson's Disease

**DOI:** 10.1002/mds.28970

**Published:** 2022-02-26

**Authors:** Olivier Rascol, Rossella Medori, Corine Baayen, Pedro Such, Didier Meulien, AMBLED Study Group

**Affiliations:** ^1^ Clinical Investigation Center CIC1436, Department of Clinical Pharmacology and Neurosciences Parkinson Expert Centre, NeuroToul Center of Excellence in Neurodegeneration (COEN) of Toulouse and NS‐Park/FCRIN Network; INSERM, University of Toulouse 3, CHU of Toulouse Toulouse France; ^2^ Prexton Therapeutics SA Geneva Switzerland; ^3^ H. Lundbeck A/S Valby Denmark

**Keywords:** dyskinesia, foliglurax, mGlu4 receptor, Parkinson's disease, positive allosteric modulator

## Abstract

**Background:**

Agents targeting the metabotropic glutamate receptor 4 have emerged as a potentially attractive new class of drugs for the treatment of Parkinson's disease (PD).

**Objective:**

The objective of this study was to evaluate the efficacy and safety of foliglurax in reducing *off* time and dyskinesia in patients with PD.

**Methods:**

This was a 28‐day, multicenter, randomized, placebo‐controlled, double‐blind clinical trial of foliglurax 10 and 30 mg as adjunct to levodopa in 157 randomly assigned patients with PD and motor complications.

**Results:**

Although dose‐dependent decreases in daily awake *off* time were apparent following treatment with foliglurax, the change from baseline to day 28 in *off* time (primary endpoint) and dyskinesia (secondary endpoint) did not improve significantly compared with placebo for either foliglurax dose. Treatment with foliglurax was generally safe, and there were no relevant safety signals.

**Conclusions:**

There was no evidence in this study that foliglurax has efficacy in improving levodopa‐induced motor complications in PD. © 2022 The Authors. *Movement Disorders* published by Wiley Periodicals LLC on behalf of International Parkinson and Movement Disorder Society

Levodopa‐induced motor complications (response fluctuations and dyskinesia) remain a common barrier to the effective management of Parkinson's disease (PD).^1^, ^2^ Despite much progress in understanding the risk factors for their development,^3^, ^4^, ^5^, ^6^ recent cohorts still report a 5‐year cumulative incidence of motor fluctuations of 29% to 54% and levodopa‐induced dyskinesia (LID) of 15% to 37%,^7^, ^8^, ^9^ increasing to 100% and 56% at 10 years.^9^ The failure of current management approaches to address the development of motor complications has resulted in a huge research effort to develop new “nondopaminergic” therapies.^10^, ^11^, ^12^

The metabotropic glutamate receptor 4 (mGlu4R) is a Gi/o protein‐coupled receptor extensively expressed in the basal ganglia and cerebellum that reduces exocytosis of neurotransmitter in response to activation by endogenous glutamate.^13^ In particular, the high levels of mGluR4 expression in the presynaptic terminals of the globus pallidus is of interest because selective activation of the receptor can inhibit γ‐aminobutyric acid and glutamate release at the striato–external pallidal and subthalamo–internal pallidal synapses, respectively.^12^, ^14^, ^15^ This is hypothesized to normalize the inhibitory/excitatory balance of the direct and indirect basal ganglia pathways, resulting in reduced motor symptoms. Foliglurax is a positive allosteric modulator of the mGlu4R that has been shown to reduce motor disability and alleviate LID in 1‐methyl‐4‐phenyl‐1,2,3,6‐tetrahydropyridine–treated primate models of PD.^16^ This proof‐of‐concept study evaluated the efficacy of 28‐day oral treatment with two doses of foliglurax compared with placebo as adjunctive therapy to levodopa to reduce the duration of *off* time. The main secondary objective was to assess the efficacy of foliglurax on reducing the severity of LID.

## Patients and Methods

### Study Design and Patients

This was a double‐blind, randomized, placebo‐controlled, parallel‐arm study of foliglurax as add‐on therapy in patients with PD^17^ (aged 35–85 years with a disease duration of ≥3 years) treated with a stable dose of levodopa (≤1600 mg/day for ≥2 weeks, or ≥6 weeks if taking a long‐acting formulation) who were experiencing both end‐of‐dose wearing off and LID. Patients had to have a Hoehn and Yahr score of 2 to 4 during the *off* period and experienced both predictable wearing‐off fluctuations and LID for ≥3 months, with ≥2 hours of *off* time and ≥2 hours of *on* time with dyskinesia per day.^18^ The LID could include troublesome and nontroublesome periods but should impact daily function (Movement Disorder Society–Unified Parkinson's Disease Rating Scale question 4.2 ≥2).^19^ Key exclusion criteria included atypical parkinsonism, significant cognitive impairment, prior neurosurgery for PD, or transcranial magnetic stimulation. Patients with clinically significant and unstable medical, surgical, or psychiatric illness were also excluded.

The study was conducted between May 25, 2017, and March 2, 2020, in accordance with the Declaration of Helsinki; study protocols were approved by the ethics committee at each of the 42 European sites, and all patients provided written informed consent. The study was registered at clinicaltrials.gov (NCT03162874).

### Treatment, Randomization, and Blinding

Following a screening period (≥28 days), patients were randomly assigned (1:1:1) using a computer‐generated sequence to 28 days of double‐blind treatment with foliglurax 10 mg (twice a day [bid], given morning and evening), 30 mg bid, or matching placebo. All patients, study site personnel, and the sponsor were blinded to group assignments. Stable concomitant medications were permitted except amantadine, apomorphine, levodopa/carbidopa intestinal gel infusion, and safinamide.

### Assessments

Patients completed a 3‐day Hauser diary^18^ (recording *off*, *on* without dyskinesia, *on* with nontroublesome dyskinesia, and *on* with troublesome dyskinesia) every 30 minutes while awake before the baseline, day 14, and day 28 visits. Dyskinesia was assessed on site using the Unified Dyskinesia Rating Scale (UDysRS)^20^ at baseline and on days 14 and 28. The timing of the UDysRS Parts 3 and 4 assessment was determined at baseline following the patient's morning levodopa dose when the patient was *on* and experiencing maximum severity dyskinesia; assessments at days 14 and 28 were performed at the same time after morning levodopa dosing.

The primary efficacy assessment was the change from baseline to day 28 in *off* time assessed by the Hauser diaries (average of 3 consecutive days). Secondary efficacy outcomes included change from baseline to day 28 in UDysRS objective (Parts 3 + 4) and total (Parts 1–4) scores and “good” *on* time (defined as *on* without troublesome dyskinesia). A responder analysis assessed the proportion of patients in each group achieving ≥1 hour reduction from baseline in *off* time. Changes from baseline to day 28 in other diary data were analyzed as exploratory measures.Blood samples for pharmacokinetic evaluation were drawn on day 1 and day 28. Pharmacokinetic analysis was conducted using noncompartmental procedures (WinNonlin version 8.1, Certara, Prince). Safety and tolerability assessments included treatment‐emergent adverse events (TEAEs), vital signs, laboratory tests, electrocardiogram.(ECG), physical examinations, and Columbia‐Suicide Severity Rating Scale.^21^

### Statistical Analyses

A total of 45 evaluable patients in each arm were calculated to provide 80% power to detect a difference of −1.2 hours in *off* time between any active treatment and the placebo group, assuming an estimated standard deviation of 2 hours with a two‐sided α‐level of 0.05. Safety was assessed for all patients who received ≥1 dose of study medication (safety set), and pharmacokinetics were assessed for all patients in the safety set who had evaluable pharmacokinetic data. Efficacy endpoints were analyzed for the full analysis set, which included all patients in the safety set who had valid baseline and ≥1 valid postbaseline assessments of *off* time. A valid diary assessment was defined as a visit that had available data for ≥2 of the 3 preceding days (each day had to have ≤4 missing diary entries [ie, ≤2 hours] per 18 hours of awake time). If valid data from only 2 days were available, the mean of these 2 days was used. Details of the mixed model for repeated measures approach to the primary and secondary efficacy analyses are provided in the supplemental information. Both doses were tested at a 5% one‐sided level without adjustment for multiplicity.

## Results

Of 233 patients screened, 157 were randomly assigned to double‐blind treatment (Figure S1). Overall, 45 of 53 patients (84.9%) randomly assigned to the foliglurax 10‐mg bid group, 48 of 52 (92.3%) patients randomly assigned to the 30‐mg bid group, and 46 of 52 (88.5%) patients randomly assigned to the placebo group completed the study. Treatment groups were generally comparable in terms of demographics and medical history, except the use of dopamine agonists was lower in the placebo than active treatment groups (55.8% with placebo vs. 68.9% and 73.1%, respectively; Table S1).

### Efficacy and Pharmacokinetics

Although least‐squares mean (LSM) differences in change from baseline to day 28 in daily awake *off* time decreased numerically with dose when compared with placebo, none of these differences were significant (Table 1, Figure S2), and the study did not meet its primary endpoint. By day 28, LSM ± SD *off* time had reduced by −0.55 ± 0.30 hours in the foliglurax 10‐mg bid group and −0.72 ± 0.31 hours in the 30‐mg bid group versus −0.29 ± 0.30 hours in the placebo group. Overall, 44.7% of patients in the 30‐mg foliglurax bid group had a decrease from baseline in daily awake *off* time of ≥1 hour compared with 31.1% in the 10‐mg bid group and 30.4% in the placebo group. However, the 90% confidence intervals (CIs) for the odds ratios between each dose level and placebo spanned 1.

**TABLE 1:** Efficacy evaluations at days 14 and 28

Efficacy outcome measurements	Placebo bid (n = 52)	Foliglurax 10 mg bid (n = 53)	Foliglurax 30 mg bid (n = 52)
Daily awake *off* time, h			
Day 14			
Change from baseline, LSM ± SE	−0.07 ± 0.26	−0.33 ± 0.26	−0.63 ± 0.28
Treatment difference versus placebo, LSM (90% CI)		−0.26 (−0.87 to 0.36)	−0.56 (−1.17 to 0.05)
Day 28			
Change from baseline, LSM ± SE	−0.29 ± 0.30	−0.55 ± 0.30	−0.72 ± 0.31
Treatment difference versus placebo, LSM (90% CI)		−0.27 (−0.96 to 0.43)	−0.44 (−1.12 to 0.25)
Daily awake good *on* time, h			
Day 14			
Change from baseline, LSM ± SE	0.41 ± 0.36	0.48 ± 0.36	0.21 ± 0.38
Treatment difference versus placebo, LSM (90% CI)		0.07 (−0.77 to 0.91)	−0.20 (−1.03 to 0.63)
Day 28			
Change from baseline, LSM ± SE	0.71 ± 0.37	0.60 ± 0.37	0.80 ± 0.39
Treatment difference versus placebo, LSM (90% CI)		−0.12 (−0.97 to 0.74)	0.09 (−0.75 to 0.93)
UDysRS total objective score			
Day 14			
Change from baseline, LSM ± SE	−2.40 ± 1.00	−3.40 ± 0.96	−2.43 ± 1.04
Treatment difference versus placebo, LSM (90% CI)		−1.00 (−3.29 to 1.29)	−0.04 (−2.34 to 2.27)
Day 28			
Change from baseline, LSM ± SE	−2.68 ± 1.04	−2.90 ± 1.01	−3.19 ± 1.08
Treatment difference versus placebo, LSM (90% CI)		−0.22 (−2.60 to 2.16)	−0.51 (−2.91 to 1.89)
UDysRS total score			
Day 14			
Change from baseline, LSM ± SE	−4.90 ± 1.62	−6.26 ± 1.56	−5.42 ± 1.72
Treatment difference versus placebo, LSM (90% CI)		−1.36 (−5.07 to 2.35)	−0.52 (−4.25 to 3.20)
Day 28			
Change from baseline, LSM ± SE	−7.51 ± 1.77	−7.49 ± 1.71	−8.11 ± 1.84
Treatment difference versus placebo, LSM (90% CI)		0.02 (−4.02 to 4.06)	−0.59 (−4.65 to 3.46)
Proportion of patients with ≥1 h reduction in *off* time			
Day 14			
n/N (%)	15/49 (30.6)	14/50 (28.0)	18/49 (36.7)
Odds ratio (90% CI)		0.85 (0.41–1.77)	1.31 (0.64–2.66)
Day 28			
n/N (%)	14/46 (30.4)	14/45 (31.1)	21/47 (44.7)
Odds ratio (90% CI)		1.00 (0.47–2.13)	1.81 (0.88–3.73)

Abbreviations: LSM, least‐squares mean; SE, standard error; CI, confidence interval; UDysRS, Unified Dyskinesia Rating Scale.

Neither foliglurax dose separated from placebo on dyskinesia as assessed by the UDysRS objective or total scores, and there were no differences (CIs included 0) between active treatment and placebo on good *on* time (Table 1) or any other Hauser diary measure (Table S2). Results of the pharmacokinetic evaluation are given in Table S3 and Figure S3; the mean geometric elimination half‐life after dosing at 10 or 30 mg bid for 28 days was 17.2 hours, and steady‐state pharmacokinetics were reached by day 14.

### Safety

TEAEs were reported for 52.8% of patients in the foliglurax 10‐mg bid group and 50.0% in the 30‐mg bid group compared with 42.3% in the placebo group (Table 2). Most TEAEs were mild or moderate in severity and resolved during the study. Seven patients had a serious TEAEs during the study, all of which were considered unrelated to treatment. The most reported TEAEs considered related to treatment were *on* and *off* phenomenon, dyskinesia, and headache. Eight patients had TEAEs that led to study withdrawal (worsening of *off*, n = 5; worsening of PD symptoms, n = 1; restlessness, n = 1; viral labyrinthitis, n = 1), of whom seven were receiving active treatment. There were no clinically relevant changes over time or differences between treatment groups with respect to clinical laboratory values, vital signs, or ECGs.

**TABLE 2:** Treatment‐emergent adverse events (TEAEs)

Safety outcome measurements	Placebo bid (n = 52)	Foliglurax 10 mg bid (n = 53)	Foliglurax 30 mg bid (n = 52)
Patients with TEAEs	22 (42.3)	28 (52.8)	26 (50.0)
Patients with treatment‐related TEAEs	11 (21.2)	12 (22.6)	6 (11.5)
Patients with serious TEAEs	3 (5.8)	3 (5.7)	1 (1.9)
Patients discontinued due to TEAEs	1 (1.9)	5 (9.4)	2 (3.8)
TEAEs reported in ≥5% of patients in any treatment group			
Fall	–	4 (7.5)	3 (5.8)
Protein urine test	1 (1.9)	1 (1.9)	3 (5.8)
*on* and *off* phenomenon	6 (11.5)	3 (5.7)	6 (11.5)
Dyskinesia	4 (7.7)	3 (5.7)	3 (5.8)
Headache	1 (1.9)	3 (5.7)	2 (3.8)

Data are provided as n (%).

## Discussion

In this proof‐of‐concept study, no significant effect on *off* time was seen with either dose of foliglurax versus placebo, and hence the study did not meet its primary endpoint. We also could not demonstrate positive results for other endpoints, including dyskinesia severity or good *on* time. Treatment with foliglurax was generally safe, and there were no relevant safety signals.

To date, the only oral antiparkinsonian treatment to have shown significant effects on both types of motor complications is a delayed‐release/extended‐release formulation of amantadine, which improved both *off* time and dyskinesia.^22^, ^23^ Following the success of amantadine, there have been numerous efforts to develop novel antiparkinsonian or antidyskinetic medications based on the glutamate hypothesis. This includes *N*‐methyl‐D‐aspartate (NMDA) antagonists (MK‐801 or memantine), α‐amino‐3‐hydroxy‐5‐methyl‐4‐isoxazolepropionic acid (AMPA) antagonists (perampanel), and Metabotropic Glutamate Receptor 5 (mGlu5) allosteric modulators (mavoglurant). None of these development programs has succeeded so far.^14^, ^24^, ^25^, ^26^ This might be explained by limitations in study designs, inadequate dosages, or inappropriate therapeutic windows to achieve efficacy without unacceptable adverse reactions.^27^ However, the negative findings of the present trial, targeting another glutamate mechanism, namely, the mGLu4 receptors, further challenges the glutamate hypothesis for the treatment of PD, reinforcing the concept that the unique effects of amantadine in this disease might be attributed to mechanisms beyond its NMDA blocking effect.^28^

A strength of this study is that it recruited patients based on having both motor fluctuations and dyskinesia. Although previous studies with amantadine have shown reductions in *off* time, patients were recruited based on their dyskinesia, and some patients may have had very little *off* time at baseline.^22^, ^23^ We also acknowledge several important limitations, including its short duration, which may have prevented us from observing the full magnitude of foliglurax effect. However, although *off* time versus baseline continued to improve (decrease) from day 14 to day 28, the treatment effect versus placebo did not change. Moreover, 28 days was considered adequate to assess the safety of foliglurax at this early stage of development and to determine early proof‐of‐concept therapeutic effect on reducing *off* time compared with placebo (current adjuncts to reduce *off* time show significant effects within this time frame). Pharmacokinetic analyses showed that plasma concentrations were in the range of what was predicted to be efficacious from the animal models^29^ with achievement of steady state by day 14, making it unlikely that a longer exposure would have provided greater efficacy.

## Financial Disclosures

Olivier Rascol has acted as a scientific advisor for drug companies developing antiparkinsonian medications (AbbVie, Adamas, Acorda, Aguettant, Alkahest, AlzProtect, Apopharma, Astrazeneca, Axvant, Bial, Biogen, Britannia, Buckwang, Centogene, Cerevel, Clevexel, Contera, Handltherapeutic, Ionis, Irlab, Lilly, Lundbeck, Lupin, Merck, MundiPharma, Neuratris, Neuroderm, Novartis, ONO Pharma, Orion Pharma, Osmotica, Oxford Biomedica, Parexel, Pfizer, Prexton Roche,Therapeutics, Quintiles, Sanofi, Servier, Sunovion, Théranexus, Takeda, Teva, Union chimique belge (UCB), and Watermark) and has received unrestricted scientific grants from academic nonprofit entities (Agence Nationale de la Recherche, Centre Hospitalo‐Universitaire (CHU) de Toulouse, France‐Parkinson, Institut National de la Santé et de la Recherche Médicale ‐Direction Générale de l'Offre de Soins (INSERM‐DHOS) Recherche Clinique Translationnelle, The Michael J. Fox Foundation, Programme Hospitalier de Recherche Clinique, and the European Commission [FP7, H2020]). Rossella Medori is currently employed by Accure Therapeutics and reports consultancy for Affiris AG within the past 12 months. Corine Baayen, Pedro Such, and Didier Meulien are employed by H. Lundbeck A/S.

## Author Roles

(1) Research Project: A. Conception, B. Organization, C. Execution; (2) Statistical Analysis: A. Design, B. Execution, C. Review and Critique; (3) Manuscript: A. Writing of the First Draft, B. Review and Critique.

O.R.: 1A, 1C, 3A, 3B

R.M.: 1A, 1C, 3B

C.B.: 1C, 2A, 2B, 2C, 3B

P.S.: 1C, 3A, 3B

D.M.: 1C, 3B

## Appendix

Following is the AMBLED Study Group member list: Olivier Rascol, Alberto Albanese, Maria Alvarez Sauco, Ernest Balaguer Martinez, Caroline Bayreuther Giordana, Stephanie Bannier, Paolo Barone, Ubaldo Bonuccelli, Placido Bramanti, Camille Carroll, Jean‐Christophe Corvol, Paola Cudia, Phillipe Damier, Luc Defebvre, Bertrand Degos, Franck Durif, Roberto Eleopra, Giovanni Fabbrini, Jorge Hernandez Vara, Jean‐Luc Houeto, Jaime Kulisevsky Bojarski, David Maltete, Maria Jose Marti Domenech, Irene Martinez Torres, Pablo Mir Rivera, Sophie Molloy, Elena Moro, Thomas Mueller, Giampietro Nordera, Christian Oehlwein, Marco Onofrj, Nicola Pavese, Tino Prell, Jason Raw, Irma Schoell, Johannes Schwarz, Klaus Seppi, Alessandro Stefani, Fabrizio Stocchi, Stephane Thobois, Claudia Trenkwalder, Richard Walker, Jurgen Winkler, and Werner Poewe.

## Supporting information


**Appendix S1:** Supporting Information

## Data Availability

The protocol and data are available upon reasonable request to the corresponding author.
