# Supplementary material for: A Randomized, Double‐Blind, Controlled Phase II Study of Foliglurax in Parkinson's Disease
Source: Mov Disord. 2022 Feb 26;37(5):1088–93. doi: 10.1002/mds.28970 (PMC9303267; doi:10.1002/mds.28970)
Supplement: Supplementary file 1 — Appendix S1: Supporting Information [file MDS-37-1088-s001.pdf]

Supplementary Appendix

Statistical analysis

The primary efficacy endpoint was analyzed using a restricted maximum likelihood-based mixed model for repeated measures (MMRM) approach, including terms for treatment, day, day-by-treatment and site as fixed factors and the baseline OFF time score and its interaction with day as covariates. An unstructured covariance structure was used to model the within-subject errors. The Kenward-Roger approximation was used to estimate denominator degrees of freedom. The analysis was performed using all available observations in the 28-day treatment period; estimated treatment differences based on least squares means between each dose of foliglurax and placebo were obtained from the MMRM at Days 14 and 28.

Secondary efficacy endpoints were analyzed in a similar fashion to the primary efficacy endpoint. Prorated UDysRS scores could be imputed in case of missing data (<8 of 15 historical and ≤5 of 11 objective items).[1]

1. Luo S, Ren X, Han W, Goetz CG, Stebbins GT. Missing Data in the Unified Dysksinesia Rating Scale (UDysRS). Movement disorders clinical practice 2018;5(5):523-526.

Figure e1. Study disposition

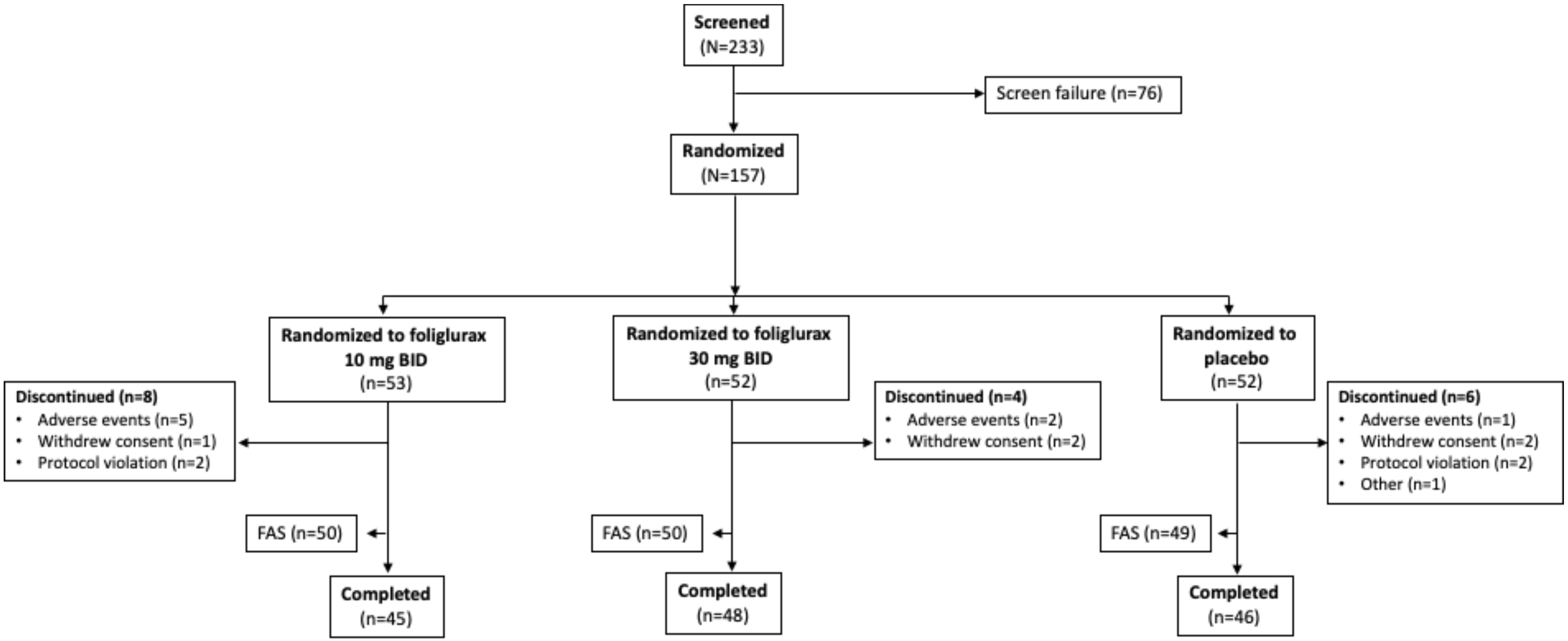

Table e1. Baseline characteristics

	Foliglurax 10mg BID (n=53)	Foliglurax 30mg BID (n=52)	Placebo BID (n=52)	Overall (n=157)
**Age, years, mean (SD)**	66 (9.2)	66 (9.1)	67 (8.9)	66 (9.0)
**Sex, n (%)**				
Male	32 (60.4%)	24 (46.2%)	28 (53.8%)	84 (53.5)
Female	21 (39.6%)	28 (53.8%)	24 (46.2%)	73 (46.5)
**Race, n (%)**				
White	40 (75.5%)	40 (76.9%)	44 (84.6%)	124 (79.0%)
Unknown*	13 (24.5%)	12 (23.1%)	8 (15.4%)	33 (21.0%)
**BMI, kg/m^2, mean (SD)**	25.2 (4.06)	25.3 (3.56)	25.0 (4.73)	25.2 (4.12)
**Time from diagnosis, years, mean (SD)**	10 (4.3)	11 (4.0)	10 (4.1)	11 (4.1)
**Modified Hoehn & Yahr (OFF), n (%)**				
2	18 (34.0%)	16 (30.8)	16 (30.8)	50 (31.8)
2.5	7 (13.2%)	8 (15.4)	4 (7.7)	19 (12.1)
3	19 (35.8%)	17 (32.7)	24 (46.2)	60 (38.2)
4	6 (11.3%)	8 (15.4)	6 (11.5)	20 (12.7)
**Levodopa, total daily dose, mg, mean (SD)**	712 (283.0)	625 (234.8)	608 (225.6)	648 (251.9)
**Concomitant PD medication, n (%)**				
Anticholinergics	3 (5.7%)	–	1 (1.9%)	4 (2.5%)
Budipine	–	1 (1.9%)	1 (1.9%)	2 (1.3%)
COMT inhibitors	8 (15.1%)	9 (17.3%)	10 (19.2%)	27 (17.2%)
Dopamine agonists	37 (69.8%)	38 (73.1%)	29 (55.8%)	104 (66.2%)
MAO-B inhibitors	22 (41.5%)	25 (48.1%)	28 (53.8%)	75 (47.8%)
**Hauser PD diary, hr, mean (SD)**				
OFF time	5.06 (2.2)	4.89 (2.1)	4.71 (2.1)	4.89 (2.1)
ON time with troublesome dyskinesia	2.24 (2.2)	2.72 (2.5)	2.70 (2.4)	2.55 (2.3)
ON time without troublesome dyskinesia	8.70 (2.5)	8.70 (2.7)	8.79 (2.7)	8.73 (2.6)
Time asleep	7.98 (2.0)	7.66 (1.8)	7.59 (1.9)	7.74 (1.9)
**UDysRS, mean (SD)**				
Total score	37 (14.1)	36 (12.2)	39 (14.0)	37 (13.4)
Total objective score	14 (8.0)	15 (6.6)	16 (8.0)	15 (7.6)

*Sites in France did not collect data on race.

Figure e2. Primary and key secondary efficacy evaluations at Day 28

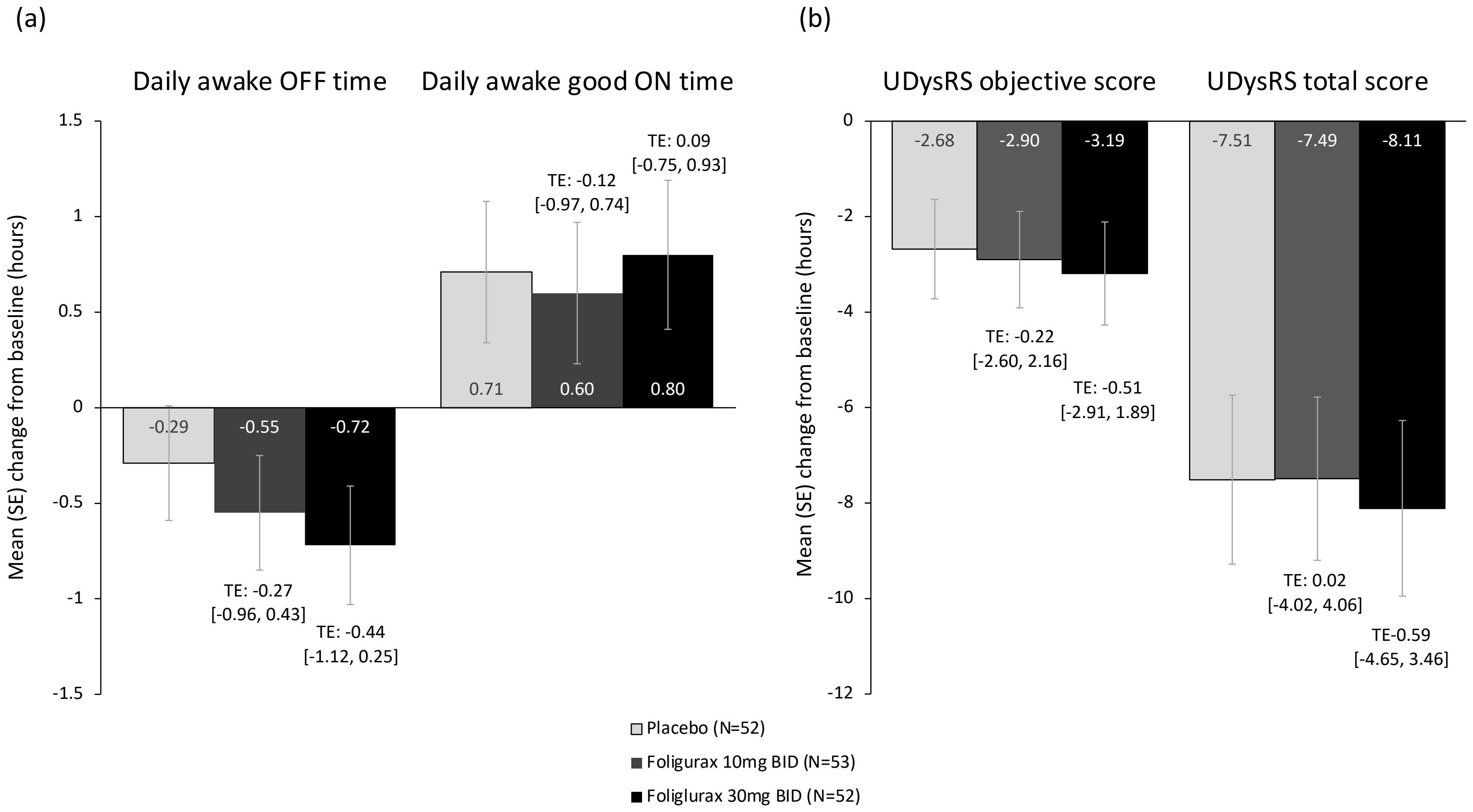

Good ON time is defined as ON without troublesome dyskinesia. TE: LS mean [90%CI] treatment effect versus placebo derived from the MMRM model including terms for treatment, day, day-by-treatment and site as fixed factors and the baseline score and its interaction with day as covariates.

Table e2. Exploratory diary outcomes at Day 28

	Placebo BID (n=52)	Foliglurax 10mg BID (n=53)	Foliglurax 30mg BID (n=52)
**Daily awake ON time, hours**			
**Day 14**			
Change from baseline, LSM ± SE	0.04 ± 0.30	0.27 ± 0.30	0.28 ± 0.30
Treatment difference vs. placebo, LSM [90%CI]		0.23 [-0.46, 0.93]	0.25 [-0.44, 0.93]
**Day 28**			
Change from baseline, LSM ± SE	0.17 ± 0.32	0.38 ± 0.32	0.44 ± 0.34
Treatment difference vs. placebo, LSM [90%CI]		0.20 [-0.54, 0.95]	0.26 [-0.47, 1.00]
**Daily awake ON time with troublesome dyskinesia, hours**			
**Day 14**			
Change from baseline, LSM ± SE	-0.36 ± 0.31	-0.25 ± 0.31	0.15 ± 0.33
Treatment difference vs. placebo, LSM [90%CI]		0.11 [-0.61, 0.83]	0.51 [-0.20, 1.23]
**Day 28**			
Change from baseline, LSM ± SE	-0.51 ± 0.33	-0.26 ± 0.34	-0.30 ± 0.35
Treatment difference vs. placebo, LSM [90%CI]		0.25 [-0.53, 1.02]	0.21 [-0.55, 0.97]

Table e3. Pharmacokinetic evaluation

Parameter	Foliglurax 10mg BID	Foliglurax 30mg BID
C_{max} (ng/mL)	2.3 (1.7)	9.8 (12.3)
t_{max} (hr)	2 (2–9)	2 (2–9)
$AUC_{0–12}$ (hr*ng/mL)	13.9 (10.0)	59.6 (72.4)
Accumulation	1.7 (0.6)	1.7 (0.6)
$t_{½}$ (hr)	17.2 (6.7)	17.2 (6.7)

Median (SD) values are shown (range for t_{max})

Figure e3. Pharmacokinetic evaluation of foliglurax (a) 10mg BID (b) 30mg BID

(a)

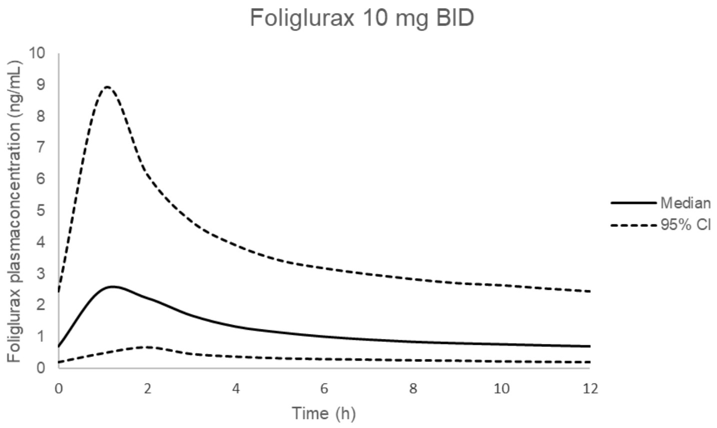

(b)

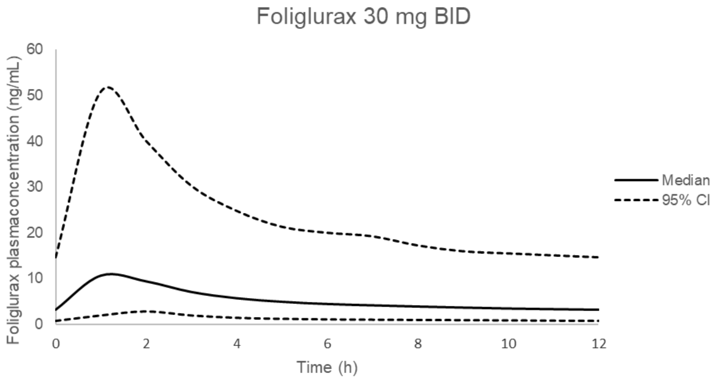

A 2- to 3-fold accumulation of foliglurax, based on $AUC_{0\text{-}8}$, was observed on Day 28. Steady state had been reached by Day 14.
